# ROS-AMPK/mTOR-dependent enterocyte autophagy is involved in the regulation of *Giardia* infection-related tight junction protein and nitric oxide levels

**DOI:** 10.3389/fimmu.2023.1120996

**Published:** 2023-03-14

**Authors:** Jingxue Wu, Yongwu Yang, Lin Liu, Weining Zhu, Min Liu, Xiran Yu, Wei Li

**Affiliations:** Heilongjiang Provincial Key Laboratory of Zoonosis, College of Veterinary Medicine, Northeast Agricultural University, Harbin, Heilongjiang, China

**Keywords:** *Giardia*, pathogenesis, autophagy, reactive oxygen species, tight junction, nitric oxide

## Abstract

*Giardia duodenalis*, a cosmopolitan noninvasive protozoan parasite of zoonotic concern and public health importance, infects the upper portions of the small intestine and causes one of the most common gastrointestinal diseases globally termed giardiasis, especially in situations lacking safe drinking water and adequate sanitation services. The pathogenesis of giardiasis is complex and involves multiple factors from the interaction of *Giardia* and intestinal epithelial cells (IECs). Autophagy is an evolutionarily conserved catabolic pathway that involves multiple pathological conditions including infection. Thus far, it remains uncertain if autophagy occurs in *Giardia*-infected IECs and if autophagic process is associated with the pathogenic factors of giardiasis, such as tight junction (TJ) barrier defects and nitric oxide (NO) release of IECs. Here *Giardia*-*in vitro* exposed IECs showed upregulation of a series of autophagy-related molecules, such as LC3, Beclin1, Atg7, Atg16L1, and ULK1, and downregulation of p62 protein. IEC autophagy induced by *Giardia* was further assessed by using autophagy flux inhibitor, chloroquine (CQ), with the ratio of LC3-II/LC3-I significantly increased and downregulated p62 significantly reversed. Inhibition of autophagy by 3-methyladenine (3-MA) rather than CQ could markedly reverse *Giardia*-induced downregulation of TJ proteins (claudin-1, claudin-4, occludin, and ZO-1; also known as epithelial cell markers) and NO release, implying the involvement of early-stage autophagy in TJ/NO regulation. We subsequently confirmed the role of ROS-mediated AMPK/mTOR signaling in modulating *Giardia*-induced autophagy, TJ protein expression, and NO release. In turn, impairment of early-stage autophagy by 3-MA and late-stage autophagy by CQ both exhibited an exacerbated effect on ROS accumulation in IECs. Collectively, we present the first attempt to link the occurrence of IEC autophagy with *Giardia* infection *in vitro*, and provides novel insights into the contribution of ROS-AMPK/mTOR-dependent autophagy to *Giardia* infection-related downregulation of TJ protein and NO levels.

## Introduction

*Giardia duodenalis*, the third most common diarrhea-causing agent worldwide, affects the health of 300 million people annually (1–3). There are eight genetic assemblages within *G. duodenalis*, among which assemblages A and B infect both human and nonhuman animals and therefore have zoonotic potential (4–6). The life cycle of the protozoan parasite *Giardia* is comprised of the disease-causing vegetative form, trophozoite, and the environmentally resistant and infective form, cyst (7, 8). Presenting symptoms of giardiasis are variable and not always evidence-based, but typically including watery diarrhea, nausea, epigastric pain, weight loss, and irritable bowel syndrome (2, 9). The complex network of *Giardia*-host interactions is implicated in the progression of giardiasis as discussed previously (10), which is far from well understood.

Autophagy is an evolutionarily conserved catabolic process necessary for degrading damaged organelles, misfolded proteins, specific signaling molecules, and even pathogens to maintain cellular homeostasis (11). Autophagy affects many cell types, besides starvation-induced bulk autophagy, it can also be induced in response to extracellular and intracellular stimuli (11). Autophagy has diverse biological functions and is characterized by the formation of a cup-shaped double-membrane vesicles known as autophagosomes as described (11). Dysregulation of autophagy has been linked to the pathogenesis of a variety of diseases related to infection, inflammation, and immunemetabolism (12). Mammalian target of rapamycin (mTOR) is a serine/threonine kinase that acts as a major inhibitory controller of autophagy (13). Upstream of mTOR is the survival phosphatidylinositol 3-kinase/protein kinase B (PI3K/AKT) pathway that modulates mTOR activity and negatively regulates autophagic process (14). The AMP (adenosine 5’-monophosphate)-activated protein kinase (AMPK) can drive autophagy activation through phosphorylation of the serine/threonine kinase Unc-51-like kinase-1 (ULK1) or through inhibition of mTORC1 pathway (15, 16). Autophagy is known to be initiated by ULK1 through phosphorylation of Beclin-1 and activation of vacuolar protein sorting 34 (VPS34) lipid kinase (17).

Autophagy is upregulated as assessed by an increase in the levels of some autophagy-related genes, such as Atg7, LC3, Atg9 and Atg12, an increase in the ratio of LC3-II to LC3-I levels, and a decrease in p62 (also known as SQSTM1) protein level (18). Accumulating data indicate a role of reactive oxygen species (ROS) in the induction of autophagy (19). Under certain pathological conditions, oxidative stress is able to activate autophagy. Autophagy, in turn, may promote reduction of oxidative damages *via* engulfing and degrading oxidized substance (20). To date, our knowledge of parasite-induced autophagy and the underlying mechanism is still poor. Helminth infections or helminth-secreted excretory/secretory (ES) products are known as the inducers of autophagy in different host cell types (21–23). In addition to helminths, infections by the protozoan parasites *Toxoplasma gondii*, *Cryptosporidium parvum*, *Entamoeba histolytica*, *Plasmodium*, and *Leishmania* also get involved in the induction of host cell autophagy (24–29;30;31). Some of the protozoans just mentioned can activate autophagy *via* an mTOR-dependent or -independent mechanism as noted (24, 27, 31). Existing evidence implicates autophagy as a candidate regulator of the pathogenesis of some of the protozoan parasites (24–26, 31). In addition, interestingly, the induction of autophagy by *Leishmania donovani* is dependent on ROS activation (29). In spite of those advances, it remains largely unexplored whether *Giardia* is able to induce intestinal epithelial cell (IEC) autophagy during noninvasive infections and whether *Giardia*-induced IEC autophagy is implicated in the pathogenesis of the parasite.

Epithelial barrier integrity have been recognized as an important part of host defense against noninvasive *Giardia* (10). *Giardia* trophozoites and their ES products have been shown to disrupt the tight junction (TJ) complexes during extracellular infections, thereby damaging intestinal barrier function and affecting nutrient absorption as noted previously (32–34). Besides, gut epithelial and immune cells can synthesize nitric oxide (NO) showing immunomodulatory and cytotoxic activities (10). It has been indicated that epithelial NO exerts a cytostatic effect on *Giardia* trophozoites and also blocks the excystation process, while the parasite can compete with IECs for the important energy source arginine and inhibit NO production in IECs (35). *Giardia*-induced arginine depletion is also known as an important inducer of IEC apoptosis (36). The collective evidence from the studies above suggests that TJ integrity and NO generation of IECs play central roles in the pathogenesis of giardiasis, however, the associated regulators remain to be elucidated. It has been demonstrated in the intestinal mucosa that autophagy participates in mucosal immune response and antimicrobial defense and maintains epithelial barrier integrity (37). Dysregulated autophagy is known to impair intestinal epithelial homeostasis and result in various pathological outcomes, such as inflammatory bowel diseases (38). It is therefore worth investigating the possible role of autophagy in regulation of *Giardia* infection-related TJ disruption and NO depletion. In reality, it has been identified that *C. parvum*-induced intestinal epithelial autophagy is involved in TJ protein destruction (31). *E. histolytica* extracts may contribute to epithelial barrier disruption through mechanisms related to autophagy and apoptosis (25). Lipopolysaccharide (LPS)-induced glial cell autophagy is able to regulate the expression of iNOS and the progress of programmed cell death (39).

*Giardia* is an important extracellular protozoan parasite responsible sometimes for acute and chronic diarrheal disease in individuals, whereas its pathogenesis is quite far from being clarified. The objectives of the present study are to explore the potential occurrence of IEC autophagy during *in vitro Giardia* infection and elucidate the associated autophagy mechanism, as well as to interpret the correlation of different stages of autophagy with *Giardia*-evoked changes in TJ protein and NO levels to reveal the potential regulatory role of autophagy in giardiasis development, for the purpose of providing novel mechanistic insights into the pathogenesis of giardiasis.

## Materials and methods

### Cell culture

The human colonic adenocarcinoma cell lines Caco-2 and HT29 cells that closely resemble normal human small IECs (40, 41), were obtained from the Cell Bank of the Chinese Academy of Sciences (Shanghai, China) and used to interact with *Giardia* trophozoites. Caco-2 cells were grown in DMEM (HyClone, Logan, UT, USA) supplemented with 10% FBS, 1% MEM NEAA, 1% GlutaMAX, and 1% penicillin/streptomycin. HT29 cells were grown in DMEM/F12 (HyClone, Logan, UT, USA) with 10% FBS and 1% penicillin/streptomycin. Cells were maintained in a 37°C incubator with a humidified atmosphere of 5% CO_2_ in air and subcultured twice a week before being seeded. The medium was changed every other day. Cells were seeded in 6-well (1 × 10^6^ cells/well), 12-well (5 × 10^5^ cells/well), 24-well (2 × 10^5^ cells/well), and 96-well (1 × 10^4^ cells/well) plates depending on the requirement of the experiments. All experiments were performed two to three days post-seeding at 80% to 90% confluence, except those concerning assessment of TJ protein levels which were performed two to three days post-confluence on fully differentiated, confluent monolayers as previously described (34).

### Parasite culture

The human-derived *G. duodenalis* WB isolate (assemblage A) was purchased from the American Type Culture Collection (ATCC 30957, Manassas, VA, USA). *Giardia* trophozoites were cultured in TYI-S-33 medium in screw-capped glass tubes incubated at 37°C as noted previously (42). Unattached or dead parasites were removed from culture tubes by medium change.

### *Giardia*-IEC interaction

After cooling in an ice bath, the parasites were harvested and washed, counted by a hemocytometer, diluted to the designated amount using cell culture medium, and used to challenge cells at a ratio of 10 parasites/cell for the indicated time periods as noted (43–48), although other challenge ratios (5 or 20 parasites/cell) have been applied before (49, 50). In time-course experiments, the trophozoites were added at different time points and cells harvested together. Before further analysis, cells were washed thrice with ice-cold PBS to remove parasites.

### qPCR analysis

Total RNA was isolated from the harvested cells cultured in 12-well plates using TRIzol reagent (Invitrogen, Carlsbad, CA, USA) and reverse-transcribed into cDNA using a HiScript II 1st Strand cDNA Synthesis Kit (Vazyme, Nanjing, China). Gene expressions of the targets LC3B, Beclin1, ULK1, Atg5, Atg7, Atg9, Atg12, Atg16L1, claudin-1, claudin-4, occludin, and zonula occludens-1 (ZO-1), were analyzed by qPCR using SYBR Green Master Mix (Vazyme, Nanjing, China) on an LC480 LightCycler system (Roche, Indianapolis, IN, USA). GAPDH was used as an endogenous control. Primers used in qPCR analysis with high efficiency were listed in **Supplementary Table 1**. The relative expression of mRNA was calculated by the use of the 2^−ΔΔCt^ method.

### Western blot analysis

Cells cultured in 6-well plates were harvested and lysed in RIPA buffer with 1% PMSF (Beyotime, Shanghai, China). Protein concentration was quantified by an enhanced BCA Protein Assay Kit (Beyotime, Shanghai, China). Western blot analysis was performed in triplicate or more. In brief, proteins were separated by 12% SDS-PAGE and electrotransferred to polyvinylidene difluoride membranes. Membranes were blocked with 5% skim milk in PBS with Tween 20 (PBST) for 2 h at room temperature (RT) and probed with the primary antibodies (1:1,000 dilution in PBST) against β-actin, LC3B, Beclin1, ULK1, Atg7, Atg16L1, p62, claudin-1, claudin-4, occludin, ZO-1, AMPK, p-AMPK, mTOR, and p-mTOR overnight at 4°C. The primary antibodies were purchased from three commercial sources (Affinity Biosciences, Changzhou, China; ABclonal, Wuhan, China; Abmart, Shanghai, China). Membranes were washed thrice with PBST and blotted with HRP-conjugated secondary antibody (1:5,000 dilution in PBST; Abmart, Shanghai, China) for 2 h at RT. Proteins were visualized using enhanced chemiluminescence detection (Syngene, Cambridge, UK). Protein band intensity was quantified by NIH Image J software (NIH, Bethesda, MD, USA).

### Immunofluorescence assay

Cells grown on cover slips in 24-well plates were fixed with 4% paraformaldehyde in PBS for 30 min at RT and permeabilized with 0.25% Triton X-100 in PBS for 10 min at RT. Nonspecific binding sites were blocked by incubation in 2% BSA in PBS for 30 min at RT. Cells were incubated with anti-LC3B antibody (dilution, 1:300) with 1% BSA in PBST overnight at 4°C, and subsequently FITC-AffiniPure goat anti-rabbit IgG (H + L) (dilution, 1:300; Jackson, West Grove, PA, USA) in the dark for 1 h at 37°C. DAPI was used to stain the cell nuclei at a concentration of 2 μg/mL (AlphaBio, Tianjin, China). The fluorescent signal was detected using a Lionheart FX Automated Microscope (BioTek, Winooski, VT, USA).

### Protein inhibition

For early-stage autophagy inhibition, cells were pretreated with 3-methyladenine (3-MA; 5 mM in use; Abmole Houston, TX, USA) for 12 h prior to being exposed to *Giardia* trophozoites. 3-MA inhibits autophagy by blocking autophagosome formation *via* the inhibition of class III PI3K (51). For autophagic flux inhibition, chloroquine (CQ; 20 μM in use; Abmole Houston, TX, USA) was applied 1 h before exposure. CQ prevents maturation of autophagic vacuoles by inhibiting fusion between autophagosomes and lysosomes (52). Rapamycin (Rapa; 500 nM in use; Abmole Houston, TX, USA), a specific inhibitor of mTOR, was applied 12 h to stimulate autophagy. ROS inhibitor N-acetyl-L-cysteine (NAC; 10 mM in use; Abmole Houston, TX, USA) was applied 2 h before exposure. A 0.1% DMSO solution, when necessary, was applied as the solvent control. Before further analysis, cells were washed thrice with PBS for drug removal.

### NO/ROS measurement

NO production represented by nitrite concentration in the supernatants of cultured cells in 96-well plates was assayed with Griess reaction using a NO Assay Kit (Beyotime, Shanghai, China). The absorbance was measured at the wavelength of 540 nm. The ROS levels in cells cultured in 24-well plates were measured using an oxidation-sensitive fluorescent probe DCFH-DA Kit, and Rosup was included as a positive control (Beyotime, Shanghai, China). The DCF fluorescence intensity in cells was measured using a Lionheart FX Automated Microscope.

### Statistical analysis

Statistical analyses were performed using the GraphPad Prism 7.0 program (GraphPad Software). The statistical significance of the differences was evaluated by the use of Student’s t-test in comparison of two groups or one-way ANOVA in comparison of three or more groups. *P*-values less than 0.05 were considered to be statistically significant (* *p* < 0.05, ** *p* < 0.01).

## Results

### *Giardia* induced autophagy in IECs

**Figure 1:**
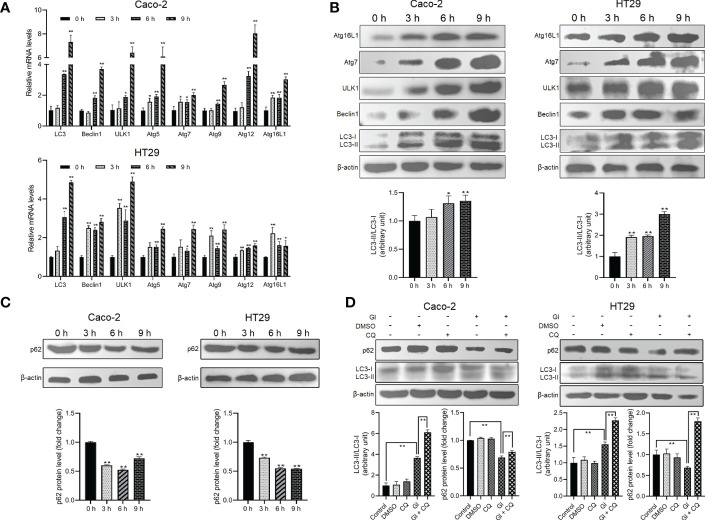
*Giardia* induces autophagy in IECs. **(A–C)** Caco-2 and HT29 cells were challenged with *Giardia* trophozoites for the indicated time periods. **(A)** The mRNA levels of several important autophagy-related genes were measured by qPCR analysis. **(B)** The proteins levels of several key autophagy-related molecules and the ratio of LC3-II/LC3-I were measured by western blot and gray value analyses. **(C)** p62 expression level was measured by western blot and gray value analyses. **(D)** Autophagy inhibition by its inhibitor CQ altered IEC autophagic response to a 6-h *Giardia* exposure as measured by western blot and gray value analyses of the ratio of LC3-II/LC3-I and p62 expression level. **(A)** Data from triplicate wells (or more) from a representative of at least three independent experiments are presented as means ± SD. *p < 0.05, **p < 0.01. **(B–D)** Images shown are representative of at least three independent experiments, and the grayscale values of the western blot bands are expressed as means ± SD. *p < 0.05, **p < 0.01. GI, *Giardia*.

As described earlier, some intracellular protozoan parasites can induce host cell autophagy. It is worthy of investigating whether *Giardia* can drive autophagic signaling during noninvasive infection of IECs. After exposure to *Giardia* trophozoites at a ratio of 10 parasites/cell for 0 h, 3 h, 6 h, and 9 h, Caco-2 and HT29 cells were examined for the changes in the mRNA and protein expression levels of some important autophagy-related molecules (**Figures 1A, B**). Markedly increased mRNA levels of LC3, Beclin1, ULK1, and Atg5/7/9/12/16L1 were observed in *Giardia*-exposed IECs within hours (**Figure 1A**). Some of the molecules were also subjected to western blot analysis, with a significant increase in the LC3-II/LC3-I ratio and protein expressions of Beclin1, ULK1, Atg7, and Atg16L1 observed, notably at 6 h and 9 h (**Figure 1B**). The ubiquitin-associated protein p62, which binds to LC3, is commonly used to monitor autophagic flux (53). *Giardia* induced a significant decrease in the protein level of p62 in Caco-2 and HT29 cells as early as 3 h after exposure (**Figure 1C**). Additionally, in both cell lines, p62 downregulation induced by a 6-h *Giardia* exposure could be recovered when CQ was used to inhibit late-stage fusion between autophagosome and lysosome (**Figure 1D**). Nevertheless, *Giardia*-induced LC3 up-expression was not influenced by inhibition of autophagic flux by CQ (**Figure 1D**). The collective data indicate that noninvasive *Giardia* is capable of inducing IEC autophagy *in vitro*.

### *Giardia*-induced autophagy regulated TJ protein expression and NO production

**Figure 2:**
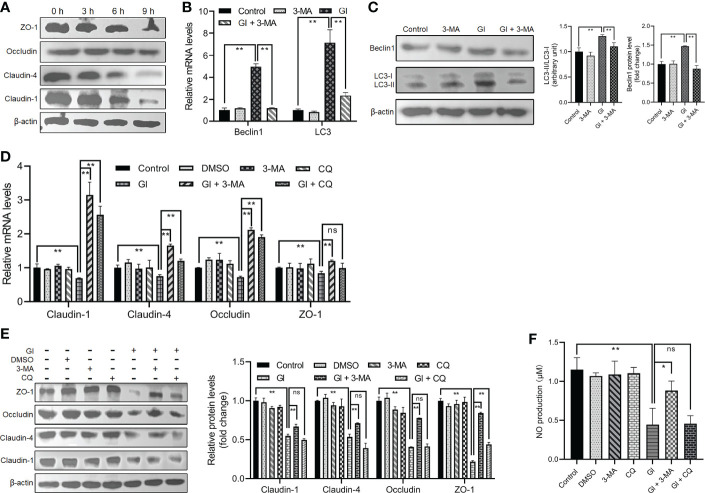
*Giardia*-induced autophagy regulated TJ protein expression and NO production in Caco-2 cells. **(A)** Western blots showing TJ protein levels at 0 h, 3 h, 6 h, and 9 h after *Giardia* exposure. **(B, C)** Caco-2 cells pretreated with 3-MA were exposed to *Giardia* trophozoites for 6 h. The mRNA and protein levels of LC3 and Beclin1 were measured by qPCR, western blot, and gray value analyses. **(D–F)** Caco-2 cells pretreated with 3-MA and CQ were exposed to *Giardia* trophozoites for 6 h. **(D, E)** The mRNA and protein levels of TJ proteins were measured by qPCR, western blot, and gray value analyses. **(F)** NO release was measured with Griess reagent method. **(A, C, E)** Images shown are representative of at least three independent experiments, and the grayscale values of the western blot bands are expressed as means ± SD. **p < 0.01. **(B, D, F)** Data from triplicate wells (or more) from a representative of at least three independent experiments are presented as means ± SD. *p < 0.05, **p < 0.01. ns, not significant; GI, *Giardia*.

As indicated earlier, various protozoan parasites are able to modulate or exploit host cell autophagy to facilitate their intracellular infectious cycle. Here we examined the potential role of autophagy induced by the extracellular parasite *Giardia* in modulating important host intestinal epithelial cell processes contributing to the pathophysiology of giardiasis such as TJ protein expression and NO production. As shown in **Figure 2A**, *Giardia* exposure led to a time-dependent downregulation of TJ protein expression (claudin-1, claudin-4, occludin, and ZO-1) in Caco-2 cells, as well as NO production as noted in a recent study (46). In addition, at 6 h after exposure, inhibition of early-stage IEC autophagy by 3-MA could suppress *Giardia*-induced upregulation of LC3 and Beclin1 as examined by qPCR, western blot, and gray value analyses (**Figures 2B, C**). We then used 3-MA and CQ to explore the potential role of autophagy in TJ/NO regulation during *Giardia*-IEC interactions. *Giardia*-induced downregulation of mRNA and protein expressions of TJ proteins (claudin-1, claudin-4, occludin, and ZO-1) and NO production in Caco-2 cells could be significantly reversed by application of 3-MA rather than CQ (**Figures 2D–F**), revealing a tight link between early-stage autophagy occurrence and *Giardia* infection-related TJ/NO downregulation.

### *Giardia*-induced autophagy signaling pathway

**Figure 3:**
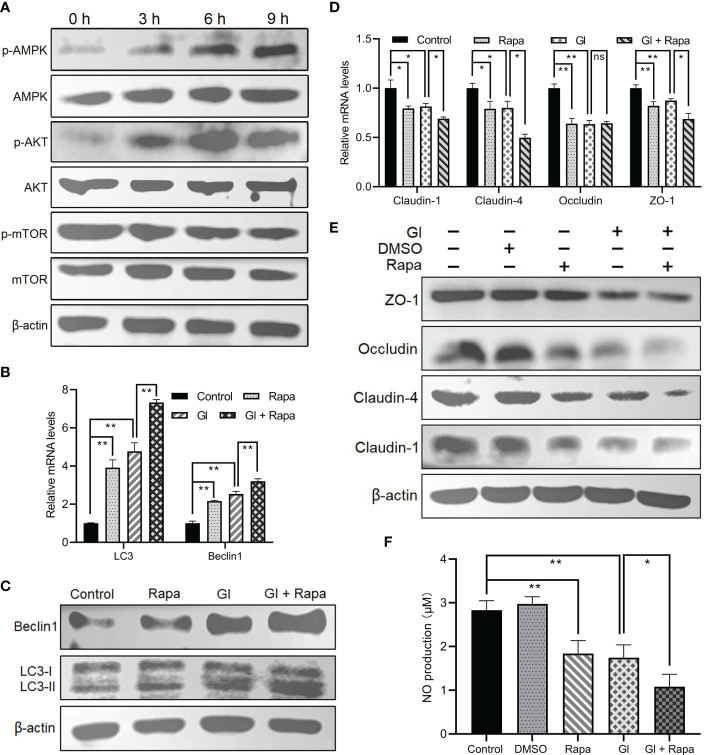
*Giardia*-induced autophagy signaling pathway in Caco-2 cells. **(A)** Western blots showing the total and phosphorylated protein levels of mTOR, AKT, and AMPK at 0 h, 3 h, 6 h, and 9 h after *Giardia* exposure. **(B–F)** Caco-2 cells pretreated with mTOR inhibitor Rapa were exposed to *Giardia* trophozoites for 6 h **(B-E)** The mRNA and protein levels of LC3, Beclin1, and TJ proteins were measured by qPCR and western blot analyses. **(F)** NO release was measured with Griess reagent method. **(A, C, E)** Images shown are representative of at least three independent experiments. **(B, D, F)** Data from triplicate wells (or more) from a representative of at least three independent experiments are presented as means ± SD. *p < 0.05, **p < 0.01. The western blots shown are representative of three independent experiments. ns, not significant; GI, *Giardia*.

It is mentioned earlier that mTOR or AKT functions as a negative regulator of autophagy, and AMPK as a positive regulator. *Giardia* exposure could prevent mTOR from becoming phosphorylated and fully active in Caco-2 cells in a time-dependent manner (**Figure 3A**), promoting the occurrence of autophagy. In addition, *Giardia* exposure led to a time-dependent dramatic increase in AMPK phosphorylation in Caco-2 cells (**Figure 3A**), which is also conductive to autophagy activation. AKT signaling seems not involved in *Giardia*-induced IEC autophagy due to the observed increasing phosphorylation of AKT following exposure (**Figure 3A**). Taken together, it could be inferred that AMPK or mTOR might participate in the regulation of *Giardia*-induced autophagy. In addition, *Giardia* exposure led to the enhanced effect on autophagy activation compared to that achieved by application of the mTOR-specific inhibitor Rapa as reflected by the levels of LC3-II/LC3-I ratio and Beclin1 expression (**Figures 3B, C**). Rapa-induced IEC autophagy and *Giardia*-induced IEC autophagy have a similar effect on promotion of downregulation of TJ protein expression and NO release (**Figures 3D–F**). Interestingly, *Giardia* exposure after mTOR inhibition by Rapa imposed a far more significant influence on inducing IEC autophagy and TJ/NO downregulation compared to that induced by *Giardia* exposure or mTOR inhibition alone (**Figures 3B–F**), implying the existence of other autophagy regulator other than mTOR, perhaps AMPK as described earlier.

### ROS-dependent activation of *Giardia*-induced autophagy

**Figure 4:**
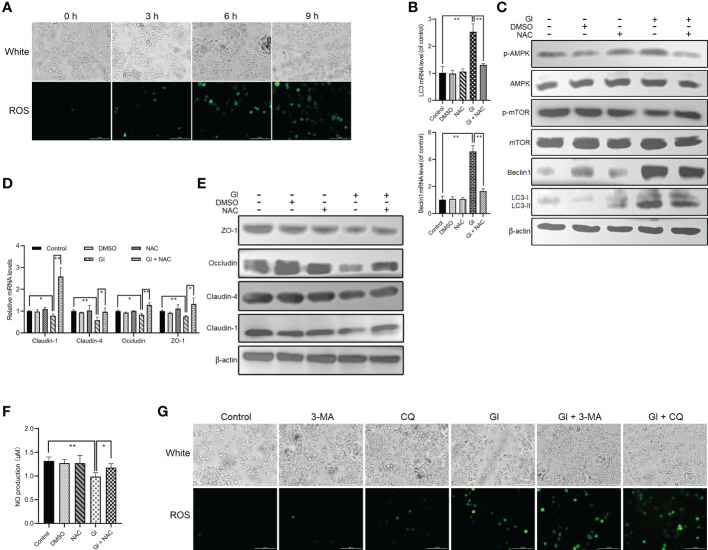
ROS-dependent activation of *Giardia*-induced autophagy in Caco-2 cells. **(A)** ROS level was examined in Caco-2 cells by fluorescence microscopy at 0 h, 3 h, 6 h, and 9 h after *Giardia* exposure (scale bar = 100 μm). **(B–F)** Caco-2 cells pretreated with ROS inhibitor NAC were challenged with *Giardia* trophozoites for 6 h **(B)** The mRNA levels of LC3 and Beclin1 were measured by qPCR analysis. **(C)** Western blots exhibiting the protein levels of LC3, Beclin1, mTOR, p-mTOR, AMPK, and p-AMPK. **(D, E)** The mRNA and protein levels of TJ proteins were examined by qPCR and western blot analyses. **(F)** NO release was measured with Griess reagent method. **(G)** Caco-2 cells pretreated with 3-MA and CQ were challenged with *Giardia* trophozoites for 6 h The level of ROS accumulation was examined by fluorescence microscopy (scale bar = 100 μm). **(A, C, E, G)** Images shown are representative of at least three independent experiments. **(B, D, F)** Data from triplicate wells (or more) from a representative of at least three independent experiments are presented as means ± SD. *p < 0.05, **p < 0.01. GI, *Giardia*.

The interconnectedness between autophagy and oxidative stress has been revealed in the past few years (54, 55). A notable ROS increase was observed following *Giardia* exposure as measured by DCF fluorescence (**Figure 4A**), which is consistent with our previous finding (43). NAC was applied to inhibit ROS generation to analyze the role of ROS in regulation of *Giardia*-induced autophagy. ROS inhibition remarkably repressed upregulated expressions of LC3 and Beclin1 and upregulated levels of LC3-II/LC3-I induced by *Giardia* as measured by qPCR and western blot analyses (**Figures 4B, C**). The results exhibited a suppression effect of ROS inhibition on IEC autophagy occurrence during *Giardia* infection. In addition, as shown in **Figure 4C**, ROS inhibition by NAC also markedly attenuated *Giardia*-induced upregulation of phosphorylated AMPK and reversed downregulation of phosphorylated mTOR, indicating the involvement of ROS in *Giardia*-activated AMPK/mTOR signaling. *Giardia*-induced TJ/NO downregulation in IECs was strikingly reversed by NAC application, demonstrating a regulatory role of ROS here (**Figures 4D–F**). Taken together, in the context of *Giardia*-IEC interactions, considering the already established close links among AMPK/mTOR signaling, *Giardia*-induced autophagy, and TJ/NO regulation, it can be inferred that *Giardia*-induced AMPK/mTOR-mediated IEC autophagy and its function in TJ/NO regulation were dependent on ROS activation. Conversely, during *Giardia*-IEC interactions, the activated autophagy exerted an inhibitory effect on ROS activation as measured by 3-MA or CQ application (**Figure 4G**).

## Discussion

**Figure 5:**
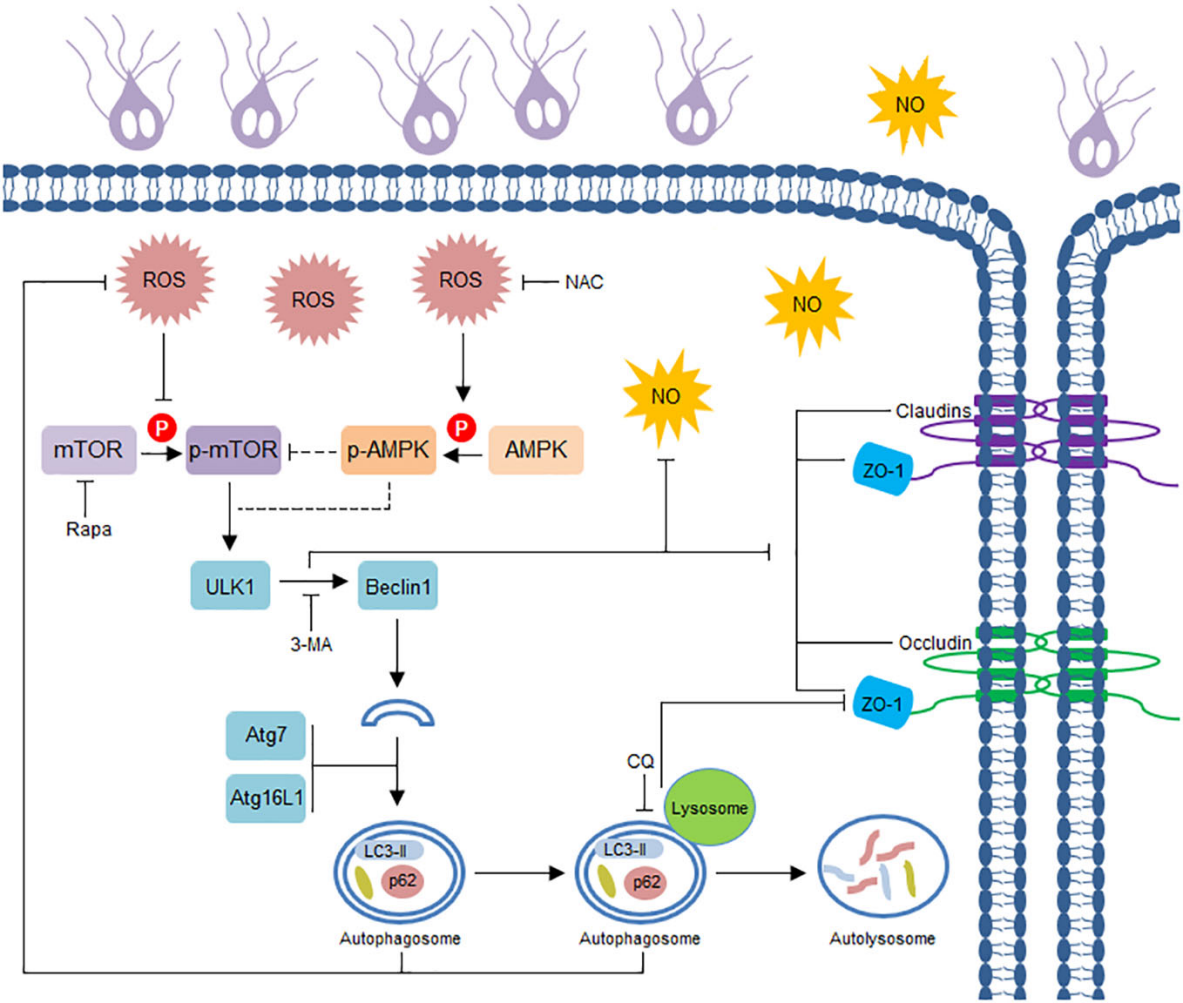
Schematic diagram illustrating *Giardia*-induced IEC autophagy pathway and its role in TJ/NO regulation.

*Giardia*, well known as a noninvasive protozoan parasite, causes a disease known as giardiasis that manifests as diarrhea and other symptoms (33), the pathogenesis of this parasite has not yet been fully elucidated. Herein, as illustrated in **Figure 5**, we initially observed that *Giardia* challenge *in vitro* activated IEC autophagy and downregulated TJ protein expression and NO production. *Giardia*-induced early-stage autophagy was then biologically linked to TJ/NO regulation in IECs, and this link was proven to be closely associated with ROS-dependent AMPK/mTOR signaling. Although *in vitro* models of interaction between *Giardia* and IECs (Caco-2 and HT29) has also been widely applied previously (46, 48, 56–59), the present observations need to be further clarified by *in vivo* studies. However, effective *in vivo* models of *Giardia* infection by WB isolate are still lacking since modeling human giardiasis in animals is complicated and difficult.

Autophagy is an important process involved in the adaptive responses to different forms of stress, including nutrient deprivation, growth factor depletion, hypoxia, and infection (60). Autophagy plays an important role in the host response to infection by multiple types of pathogens, including bacteria, viruses, protozoa, and parasitic helminths (27, 28, 61–65). Some intracellular protozoan parasites can manipulate host autophagy in order to escape cellular elimination or maintain infection within a host. *T. gondii* induces host cell autophagy to enhance its proliferation through a mechanism involving calcium rather than mTOR (24). *C. parvum*-induced mTOR-dependent autophagy is shown to modulate host cell processes related to pathophysiology of cryptosporidiosis (31). *L. donovani* -induced autophagy is associated with ROS activation and PI3K/AKT or ERK/MAPK signaling, which influences the engulfment of neutrophils by the macrophages (29). Autophagy machinery of host cells can be hijacked by apoptotic-like *Leishmania* to reduce T-cell proliferation and T-cell-mediated parasite elimination (26). It has also been revealed that peroxiredoxin of *E. histolytica* could induce autophagy and cytotoxic effects in macrophages 30). Here we demonstrated a ROS-AMPK/mTOR-dependent autophagy machinery induced by extracellular *Giardia*. In reality, it has become well known that oxidative stress operate as the converging point of autophagic stimuli and ROS as an important intracellular signal transducer that sustains autophagy (66). Autophagy, in turn, is able to alleviate oxidative damage and ROS accumulation by removing protein aggregates and damaged organelles like mitochondria (67). In the present study, *Giardia*-induced autophagy had an inhibitory effect on the levels of ROS in IECs. Considering the promoting role of *Giardia*-induced ROS accumulation in autophagy occurrence in IECs we established earlier here, it can be inferred that the interrelation between ROS and autophagy during *Giardia*-IEC interactions could be a potential mechanism for maintaining host homeostasis, possibly favoring the development of chronic infection.

*C. parvum* has been known as an inducer of dysregulation of intestinal ion transport and barrier function, which could be involved or result in diarrhea (68, 69). *C. parvum*-induced IEC autophagy is shown in relation to TJ regulation (31). *Giardia* and its secreta contribute to intestinal epithelial barrier disruption, which involves immune modulation and pathogenesis of giardiasis (33, 34). Our study provided the first evidence demonstrating that *Giardia*-induced TJ protein downregulation was correlated to the regulatory role of autophagy. Autophagy was reported to regulate intestinal barrier function *via* inducing lysosomal degradation of claudin-2, thereby affecting epithelial permeability (70). An increasing amount of studies have also identified a vital role for autophagy and more specifically, lysosomal function, in maintaining intestinal barrier and mucosal homeostasis (38). Nevertheless, our study proved early-stage autophagy as an important contributor to TJ regulation in IECs exposed to *Giardia*, rather than autophagic flux or lysosomal activity. In any case, extreme caution should be exercised in the application of 3-MA for studying early-stage autophagy due to the differential temporal effects on class I and class III PI3K as noted (71), calling for the use of additional inhibitor in parallel with 3-MA in future studies. It has been evident that Beclin1 functions as an essential regulator for intestinal epithelial TJ barrier (72). However, the specific mechanism by which early-stage autophagy promotes *Giardia*-induced TJ protein downregulation is not clear, perhaps in relation to activation of specific or unique autophagy genes. In addition, further *in vitro* and *in vivo* studies are needed to elucidate the mechanism of transcriptional induction of autophagy genes and TJ genes during *Giardia* infection, in order to further clarify the involvement of *Giardia*-induced autophagy or transcription of autophagy genes in the change in TJ protein expression. Actually, a recent study has unveiled that Atg8a plays an important nuclear role in the regulation of autophagy gene expression in *Drosophila*, involving its acetylation status and interaction with transcription factor Sequoia (73).

It has been proven that NO is cytostatic rather than cytotoxic for *Giardia* trophozoites (35). *Giardia* infections downregulate the expression of iNOS in IECs (46, 74). Likewise, downregulated iNOS levels are present in *Giardia*-infected paediatric patients (75). In contrast, upregulated iNOS expression was observed in mice and humans infected with *Giardia* (76–78), although not necessarily functional. It is also known that *Giardia* might circumvent NO-mediated host defense by competing with IECs for arginine uptake (10, 35, 36). Rapa-induced mTOR inhibition reveals a regulatory role for iNOS expression in macrophages (79). LPS-induced autophagy has been shown to suppress the expression of iNOS in glial cells (39). In the present study, *Giardia*-induced downregulation of NO production in IECs was shown to be promoted by the activation of early-stage autophagy, while the underlying mechanism needs to be further explored.

Autophagy and apoptosis are biologically linked as discussed previously (80). Although our recent studies have demonstrated *Giardia*-induced TNFR1-mediated extrinsic and ROS-mediated mitochondrial pathways of apoptosis in Caco-2 and HT29 cells (43, 44, 48), the interplay between autophagy and apoptosis in IECs, notably primary epithelial cells, during noninvasive *Giardia* infections is still unknown and is not elucidated under the present circumtances, and therefore needs to be further investigated.

In conclusion, this study demonstrated that, in response to *Giardia* infection, IECs expressed a ROS-AMPK/mTOR-mediated autophagy machinery. Additionally to that, *Giardia*-induced IEC TJ protein downregulation and NO release reduction were specifically linked to the regulatory function of early-stage autophagy rather than autophagic flux. Nevertheless, further research and efforts are needed to identify *Giardia*-secreted toxins or specific virulence factors related to IEC autophagy initiation and to clarify the specific autophagic factors responsible for *Giardia* infection-related TJ/NO regulation. In addition, the *in vitro* interaction system used in this study could not present complexity of interactions from neighboring cells of different types and functions. Therefore, a further functional barrier assessment derived from the application of organoid-derived monolayers and/or laboratory animal models is required to fully understand the involvement of ROS-AMPK/mTOR-mediated early-stage autophagy in the TJ loss from *Giardia*-infected epithelia.

## Data availability statement

The original contributions presented in the study are included in the article/**Supplementary Material**. Further inquiries can be directed to the corresponding author.

## Author contributions

JW and WL conceptualized the manuscript. JW, LL, WZ, ML, and XY performed the experiments. JW and YY analyzed the data and elaborated the figures. JW and WL drafted and revised the manuscript. All authors contributed to the article and approved the submitted version.
