# Supplementary material for: ROS-AMPK/mTOR-dependent enterocyte autophagy is involved in the regulation of Giardia infection-related tight junction protein and nitric oxide levels
Source: Front Immunol. 2023 Mar 14;14:1120996. doi: 10.3389/fimmu.2023.1120996 (PMC10043474; doi:10.3389/fimmu.2023.1120996)
Supplement: Supplementary file 1 [file Table_1.pdf]

Table S1 Primer pairs used in qPCR analysis.

Gene	Accession no.	Primer (5′ to 3′)	Product size
LC3B	NM_022818.5	F: CAGGAGGCATTGCTGATGAT	167 bp
		R: GAAGGCTGGGGCTCATTT	
Beclin1	NM_003766.5	F: ACCTCAGCCGAAGACTGAAG	165 bp
		R: AACAGCGTTTGTAGTTCTGACA	
ULK1	NM_003565.4	F: GGCAAGTTCGAGTTCTCCCG	97 bp
		R: CGACCTCCAAATCGTGCTTCT	
Atg5	NM_001286108.2	F: AAAGATGTGCTTCGAGATGTGT	144 bp
		R: CACTTTGTCAGTTACCAACGTCA	
Atg7	NM_001349234.2	F: ATGATCCCTGTAACTTAGCCCA	114 bp
		R: CACGGAAGCAAACAACTTCAAC	
Atg9	NM_024085.5	F: TGTTTCTCAATGAATGGAGCCTC	109 bp
		R: AAGTTAGCGATGCCAATCCAC	
Atg12	NM_004707.4	F: TAGAGCGAACACGAACCATCC	153 bp
		R: CACTGCCAAAACACTCATAGAGA	
Atg16L1	NM_198890.3	F: ATGCGCGGATTGTCTCAGG	138 bp
		R: GTCCACTCATTACACATTGCTCT	
Claudin-1	NM_021101.5	F: CCTCCTGGGAGTGATAGCAAT	145 bp
		R: GGCAACTAAAATAGCCAGACCT	
Claudin-4	NM_001305.5	F: GGGGCAAGTGTACCAACTG	109 bp
		R: GACACCGGCACTATCACCA	
Occludin	NM_002538.4	F: CGGCGAGTCCTGTGATGAG	119 bp
		R: TCTTGTATTCCTGTAGGCCAGT	
ZO-1	NM_001330239.4	F: ACCAGTAAGTCGTCCTGATCC	128 bp
		R: TCGGCCAAATCTTCTCACTCC	
GAPDH	NM_001357943.2	F: CAGGAGGCATTGCTGATGAT	138 bp
		R: GAAGGCTGGGGCTCATTT	
